# Prognostic Role of Survivin in Bladder Cancer: A Systematic Review and Meta-Analysis

**DOI:** 10.1371/journal.pone.0076719

**Published:** 2013-10-18

**Authors:** Chanhoo Jeon, Myong Kim, Cheol Kwak, Hyeon Hoe Kim, Ja Hyeon Ku

**Affiliations:** Department of Urology, Seoul National University College of Medicine, Seoul, Korea; Biomedical Research Foundation, Academy of Athens, Greece

## Abstract

**Purpose:**

The objective of the present study was to conduct a systematic review and meta-analysis of published literature investigating the survivin expression and its effects on bladder cancer prognosis.

**Materials and Methods:**

We carefully searched online Pubmed, Cochrane Library and SCOPUS database from August 1997 to May 2013.

**Results:**

A total of 14 articles met the eligibility criteria for this systematic review. The eligible studies included a total of 2,165 patients with a median number of 155 patients per study (range: 17–726). Of the 14 studies, nine evaluated immunohistochemistry in formalin-fixed paraffin-embedded tissue blocks. In non-muscle invasive bladder tumor, the pooled hazard ratio (HR) was statistically significant for recurrence-free survival (pooled HR, 1.81; 95% confidence interval [CI], 1.30–2.52), progression-free survival (pooled HR, 2.12; 95% CI, 1.60–2.82), cancer-specific survival (pooled HR, 2.01; 95% CI, 1.32–3.06), and overall survival (pooled HR, 1.53; 95% CI, 1.02–2.29). The overall HRs by survivin status were robust across advanced stages. When only adjusted survival data were included, statistically significant differences were identified for all survival subgroup analyses. There was no between-study heterogeneity in the effect of survivin status on the majority of meta-analyses. There was no clear evidence of publication bias in this meta-analysis.

**Conclusions:**

Survivin expression indicates worse prognosis in patients with bladder cancer but the results should be interpreted with caution. It is necessary that better-designed studies with standardized assays need to provide a better conclusion about the relationship between survivin expression and the outcome of patients with bladder cancer.

## Introduction

Bladder cancer is the second most common cancer arising in the genitourinary tract [1], and is characterized by its variable prognosis. In about 70% of patients with non-muscle invasive bladder cancer, tumors recur and some of these patients will eventually show progression towards muscle invasive cancer. Tumors that are muscle invasive have a high risk of progression, despite radical cystectomy and other treatments. One of important focuses in bladder cancer research is the prediction of tumor recurrence and tumor progression. Conventional prognostic factors, like tumor stage and grade, do not accurately predict the clinical outcome of many patients with bladder cancer, because of the inherent heterogeneity of tumor biology and patient characteristics. Additional effective biomarkers are required for explaining variability of outcome in patients with bladder cancer.

The ability of molecular markers to predict recurrence and progression of the disease, response to treatment, and survival has been investigated intensively over the last decades. Although numerous potential bladder tumor markers have been identified, their significance remains controversial. Survivin has been described as the smallest, structurally unique member of the ‘inhibitor of apoptosis’ family [2]. As compared with normal differentiated adult tissues, survivin is frequently overexpressed in tumors [3]. Functionally, survivin displays regulatory functions for control of cell division and inhibition of apoptosis, induces angiogenesis, and plays a pivotal role in cancer progression [4]. Because of this upregulation in malignancy and its functional involvement in apoptosis, as well as proliferation, survivin is attracting considerable interest as a potential cancer biomarker [5]. Generally, high survivin mRNA or protein expression is correlated with aggressive behavior of tumor cells, and survivin expression has been established as a prognostic factor in several tumor types [6]–[8].

Thus, in urothelial carcinoma of the urinary bladder, survivin has been suggested as a promising biomarker for cancer prognosis. Survivin expression has been reported to be indicator of poor prognosis in bladder cancer, whereas some other studies did not show the same results [9]–[11]. Because reports about its prognostic significance in bladder cancer are comparatively few, the combination of these data to reach a reasonable conclusion is fairly necessary at present. The objective of the present study was to conduct a systematic review and meta-analysis of published literature investigating the survivin expression and its effects on bladder cancer prognosis. We also aimed to assess the quality of published studies.

## Materials and Methods

### Search Strategy and Selection Criteria

We carefully searched online Pubmed, Cochrane Library and SCOPUS database. Since the first survivin article was published in 1997, we searched literatures published from August 1997 to May 2013, to identify relevant studies by combining the keywords [survivin] AND [urinary bladder neoplasms] OR [urinary AND bladder AND neoplasms] OR [bladder AND cancer] OR [bladder cancer]. To be eligible for our meta-analysis, studies had to be English-language published documents dealing with histopathologically confirmed bladder cancer at the time of study inclusion.

The inclusion criteria for our systematic review were, as follows: (i) articles were published in English in the periodical literature; (ii) the histologic type of the tumors was urothelial carcinoma; (iii) expression of the survivin was evaluated in tissues or urines; (iv) the association between survivin expression levels and survival outcome was investigated; and (v) the authors offered the size of the sample, hazard ratios (HRs) and their 95% confidence intervals (CIs) or other information that could help infer the survival results in the paper. When multiple articles were published by the same authors or group, the most recently published or most informative single article was selected to avoid duplication of the patient data. Duplicate reports were included in the specific analyses only if they performed different subgroup analyses. No attempt was made to restrict the search according to more specific methodological characteristics. Accordingly, the following exclusion criteria were used: (i) review articles or letters to the editor; (ii) laboratory studies, such as studies on bladder cancer cell lines and animal models; and (iii) studies which did not provide sufficient data to acquire HR and its standard error.

To minimize the bias and to improve reliability, two independent reviewers (C.W.J and J.H.K.) assessed the eligibility of abstracts identified by the search. If studies seemed appropriate, the full manuscript was scrutinized and the study was deemed “relevant” if it met the inclusion criteria. If the eligibility was unclear from the abstract, the full article was retrieved for clarification. The full text publication was independently screened by two of the authors (C.W.J and J.H.K.). Disagreements between reviewers were resolved by consensus.

### Data Extraction and Quality Assessments

The extracted data elements of this review included the following: (i) publication details: country, first author’s last name, publication year, period of recruitment, and study design; (ii) characteristics of the studied population: sample size, mean or median age, gender, inclusion and exclusion criteria, tumor characteristics, treatment, endpoint definition, and follow-up period; (iii) cut-off value of positive expression and the antibodies used for immunohistochemistry (IHC), as well as biologic samples and the type of measurements used to determine survivin status; and (iv) survival curves, the exact data of total and exposed number in case and control groups, as well as HRs and their CIs.

Study quality was assessed independently by two investigators (C.W.J and J.H.K.). Any disagreement was resolved by discussion. Although no standard quality assessment method is currently available, an assessment of study methodology was made according to previously defined criteria. We systematically assessed the quality of all included studies using the predefined form by De Graeff et al [12], which was adapted from Hayes et al [13] and McShane et al [14]. Briefly, the following criteria were investigated: (i) the study reported inclusion and exclusion criteria; (ii) study data were prospectively or retrospectively gathered; (iii) clinical and pathological characteristics of the patients were sufficiently described; (iv) the assay used was sufficiently described; (v) a definition of the study endpoint was provided; (vi) the follow-up time was described; and (vii) the study reported how many patients were lost to follow-up or were not available for statistical analysis.

### Statistical Analysis

#### Primary analysis

The recommended summary statistics for meta-analysis of time-to-event data are the logHR and its variance, which account for both the time it takes for an event to occur, as well as censoring. For each trial, this HR was estimated by a method depending on the data provided in the publications. The simplest method consisted in the direct collection of HR and their 95% CI from the original article. If those data were not available, previously reported indirect methods were utilized for extracting the logHR and variance, due to the paucity of prognostic literature directly reporting these values [15]–[17]. A random-effect model was used to obtain the summary HRs and 95% CIs. An observed HR >1 indicated worse outcome for the study group relative to the reference group, and would be considered statistically significant if the 95% CI did not overlap, with p<0.05.

#### Subgroup analysis

Subsequently, we assessed the effect of unadjusted HR on the survivin results in patients with non-muscle invasive bladder tumor. First, attempt was made to use only adjusted survival data as part of this meta-analysis. Studies that did not report an adjusted HR for survival after controlling for potential confounding clinical variables in a multivariable analysis (e.g. Cox regression analysis including important clinical factors, such as age, grade, and/or performance status) were excluded, since the accuracy of HRs estimated from Kaplan-Meier survival curves without a multivariate analysis was uncertain [18]–[20]. These data were applied in a subgroup, and meta-analyses were performed to test the stability of our conclusions.

#### Sensitivity analysis

We performed sensitivity analyses in patients with non-muscle invasive bladder tumor. Through sensitivity analyses, we examined if our pooled estimate of the prognostic value of survivin status was largely influenced by the method for determination of survivin expression. Studies using immunohistochemical (IHC) expression were included in sensitivity analyses.

#### Assessment of heterogeneity

Heterogeneity was assessed using the chi-square test for heterogeneity, with a p value of <0.05 taken to reflect the presence of significant heterogeneity [21]. The I^2^ statistic was calculated to quantify the degree of heterogeneity [22].I^2^ describes the proportion of total variation in meta-analysis estimates, which is due to inter-study heterogeneity, rather than sampling error, and is measured from 0% to 100%, with increasing I^2^ values indicating a larger effect of between-study heterogeneity in the meta-analysis.

#### Publication bias

For those meta-analyses including 10 or more studies, we assessed the possibility of publication bias. Publication bias was evaluated using the funnel plot. In the absence of bias, the graph should resemble a symmetrical inverted funnel; conversely, in the presence of bias, the plot should appear skewed and asymmetrical.

The meta-analysis was undertaken using Review Manager (RevMan) software version 5.0 (RevMan 5; The Nordic Cochrane Center, The Cochrane Collaboration, Copenhagen, Denmark).

## Results

**Figure 1:**
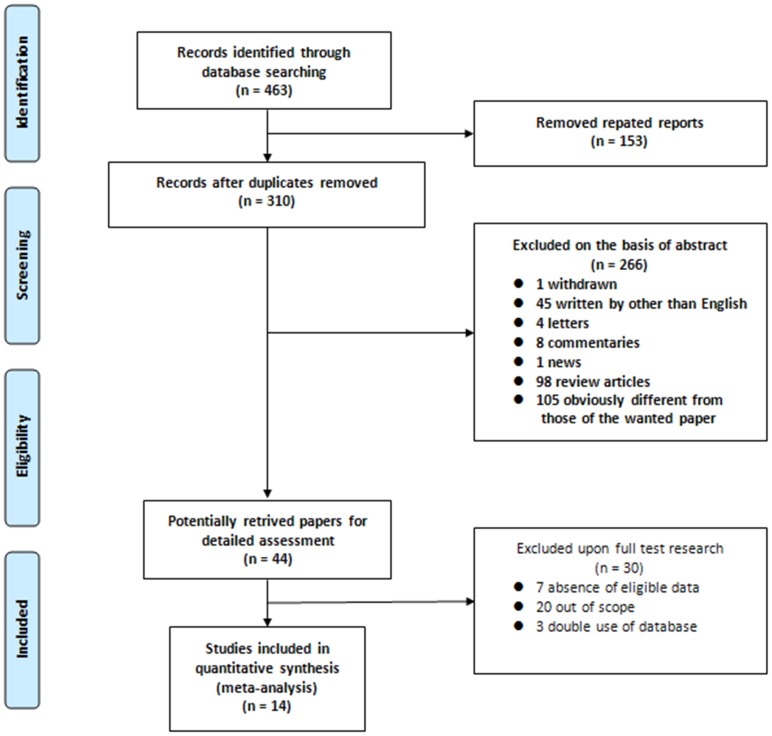
Methodological flow chart of the systematic review.

Our search strategy identified 463 articles. Following deduplication, two reviewers independently screened the identified titles and abstracts. They subsequently agreed that 44 articles should be retrieved for detailed review; for these manuscripts, full texts were obtained. On careful review of study methodologies, 31 were excluded for the following reasons: 20 studies had no formal investigation of outcomes [23]–[42]. Instead, these studies assessed only the predictive ability and included the detection validity in the diagnosis of bladder cancer or based their results on association tests; seven studies provided incomplete information for HRs and 95% CIs [43]–[49]; and three studies were excluded because it contained duplicate data [9], [50], [51]. Thus, a total of 14 articles met the eligibility criteria for this systematic review [10], [11], [52]–[63]. A flow diagram of the study selection process is presented in Fig. 1.

Table 1 outlines the main characteristics of the included studies. Considering the selected studies, one was carried out in the United States, nine in Europe, three in Asia, and one was multinational. None of selected studies was prospective study. Patient tissues were the mostly common samples used to detect survivin, but in two studies [54], [57] the authors used urine specimens to assess survivin mRNA. Four (44.4%) of nine evaluated IHC staining in formalin-fixed paraffin-embedded tissue blocks did not define the primary antibody used [55], [56], [61], [62]. A wide range of dilutions was used (1/50 to 1/1,600). The definition of survivin overexpression also varied among studies. The cutoff value used to define survivin overexpression was 10% in most studies, whereas in the remaining two studies, the cut-off value was 8% and 20%, respectively [55], [59]. Immunopositive cells were defined according to the percentage of nuclear [55], [58], [60], cytoplasmic [53] or both [56], [59], [62], [63] staining. Four studies documented whether staining assessment was blinded to outcome status [53], [58], [60], [61]. The median quality score was recorded as 5 (range: 3–6). There was no significant correlation between study size and quality scores (Spearman’s r = 0.472, p = 0.210).

**Table 1:** Main characteristics of the eligible studies.

Study	Year	Country	Recruitment period	Study design	Inclusion and exclusion criteria	Consecutive Patients	Specimen	Method	Compartment	Cut-off	Definitionof survival	Blindassessment	Quality Assessment(0–8)
Gazzaniga^10^	2003	Italy	1996–1998	retrospective	no	NA	tissue	RT-PCR	−	−	yes	NA	5
Schultz^52^	2003	Netherlands	NA	retrospective	no	NA	tissue	real-time RT-PCR	−	0.26*	yes	NA	5
Ku^53^	2004	Korea	1993–1997	retrospective	no	no	tissue	IHC	cytoplasm	20%	yes	blind	5
Schultz^54^	2004	Netherlands	NA	retrospective	no	NA	urine	real-time RT-PCR	−	0.13*	yes	NA	3
Yin^55^	2006	China	NA	retrospective	no	yes	tissue	IHC	nuclear	8%	no	NA	4
Karam^56^	2007	USA	1995–2003	retrospective	no	NA	tissue	IHC	nuclear or cytoplasm	10%	yes	NA	3
Pina-Cabral^57^	2007	Portugal	NA	retrospective	no	NA	urine	RT-PCR	−	−	yes	NA	3
Skagias^58^	2009	Greece	1998–2005	retrospective	no	NA	tissue	IHC	nuclear	10%	yes	blind	5
Weiss^59^	2009	Germany	1982–2004	retrospective	no	no	tissue	IHC	nuclear or cytoplasm	20%	yes	NA	5
Gradilone^11^	2010	Italy	NA	retrospective	yes	NA	tissue	RT-PCR	−	−	no	NA	4
Fristrup(Denmark)^60^	2012	Denmark	1979–2007	retrospective	no	NA	tissue	IHC	nuclear	10%	yes	blind	5
Fristrup(validation1)^60^	2012	Sweden	1984–2005	retrospective	no	NA	tissue	IHC	nuclear	10%	yes	blind	5
Fristrup(validation2)^60^	2012	Spain	1994–2008	retrospective	no	NA	tissue	IHC	nuclear	10%	yes	blind	5
Xi^61^	2013	China	2000–2006	retrospective	yes	no	tissue	IHC	NA	10%	yes	blind	6
Shariat^62^	2009	Multination	1983–2005	retrospective	yes	no	tissue	IHC	nuclear or cytoplasm	10%	yes	NA	4
Als^63^	2007	Denmark	1995–2004	retrospective	yes	NA	tissue	IHC,microarray	cytoplasm with an intensity of 2 or 3	10%	no	NA	6

* survivin mRNA copy number/cyclophilin mRNA copy number.

NA: not available, RT-PCR: reverse transcriptase-polymerase chain reaction, IHC: immunohistochemistry.

The 14 eligible studies included a total of 2,165 patients, with a median number of 155 patients per study (range: 17–726). Basic sociodemographic information, such as sex and age, was missing from 28.6% and 28.6% of studies, respectively. Other characteristics such as the patient and tumor characteristics are summarized in the Table S1 and S2 (in File S1). Of the 1,755 patients available in the present study, survivin overexpression was detected in 846 (48.2%). There were higher frequencies of survivin overexpression with tumor grade were higher. However, no relationship was found between survivin expression and T stage (Table S3 in File S1).

Table 2 summarizes the methods for estimation of HR. nine (64.3%) studies reported the cofactors used in the multivariate models, which varied widely, even for a given endpoint. Twenty-three clinicopathologic factors were incorporated in one or more of the included studies’ multivariate analyses. The most common cofactors in the studies that used multivariate analysis to assess the risk of mortality were grade (n = 6) and pT stage (n = 6).

**Table 2:** Estimation of the hazard ratio.

Study	Survival analysis	HR estimation	Co-factors	Analysis results
Gazzaniga^10^	recurrence-free	p value, event number (univariate)	−	not significant
Schultz^52^	recurrence-free	absence of eligible data	−	significant
	progression-free	p value, event number (univariate)	−	not significant
	cancer-specific	absence of eligible data	−	not significant
Ku^53^	recurrence-free	HR, 95% CI (multivariate)	age, sex, size, number, architecture, grade, T stage	significant
Schultz^54^	recurrence-free	p value, event number (univariate)	−	significant
Yin^55^	progression-free	HR, 95% CI (multivariate)	age, grade, T stage, grade and stage, ki67, BIRC5-C	significant
	cancer-specific	HR, 95% CI (multivariate)	age, grade, T stage, grade and stage, ki67, BIRC5-C	significant
Karam^56^	recurrence-free	HR, 95% CI (multivariate)	grade, T stage, intravesical therapy	significant
	progression-free	HR, 95% CI (multivariate)	grade, T stage, intravesical therapy	significant
	cancer-specific	HR, 95% CI (multivariate)	grade, T stage, intravesical therapy	not significant
Pina-Cabral^57^	recurrence-free	p value, event number (univariate)	−	significant
Skagias^58^	recurrence-free	HR, 95% CI (multivariate)	grade, T stage	Significant
	overall	HR, 95% CI (multivariate)	grade, T stage	not significant
Weiss^59^	recurrence-free	p value, event number (univariate)	−	significant
	progression-free	p value, event number (univariate)	−	not significant
	cancer-specific	p value, event number (univariate)	−	not significant
Gradilone^11^	recurrence-free	HR, 95% CI (multivariate)	circulating tumor cell	not significant
Fristrup (Denmark)^60^	progression-free	HR, 95% CI (multivariate)	cathepsin E, maspin, PIK1	significant
	cancer-specific	HR, 95% CI (multivariate)	cathepsin E, maspin, PIK1	significant
	overall	HR, 95% CI (multivariate)	cathepsin E, maspin, PIK1	significant
Fristrup (validation)^60^	progression-free	HR, 95% CI (multivariate)	cathepsin E, maspin, PIK1	significant
Xi^61^	progression-free	HR, 95% CI (multivariate)	grade, T stage, livin	significant
Shariat^62^	recurrence-free	HR, 95% CI (multivariate)	Age, sex, grade, pT stage, N stage, surgical margin, LVI,concomitant CIC, ACH	significant
	cancer-specific	HR, 95% CI (multivariate)	Age, sex, grade, pT stage, N stage, surgical margin, LVI,concomitant CIC, ACH	significant
Als^63^	overall	HR, 95% CI (multivariate)	visceral metastasis, emmprin	significant

HR: hazard ratio, CI: confidence interval, BIRC5-C: cytoplasmic staining of survivin, LVI: lymphovascular invasion, CIS: carcinoma in situ, ACH: adjuvant chemotherapy.

**Figure 2:**
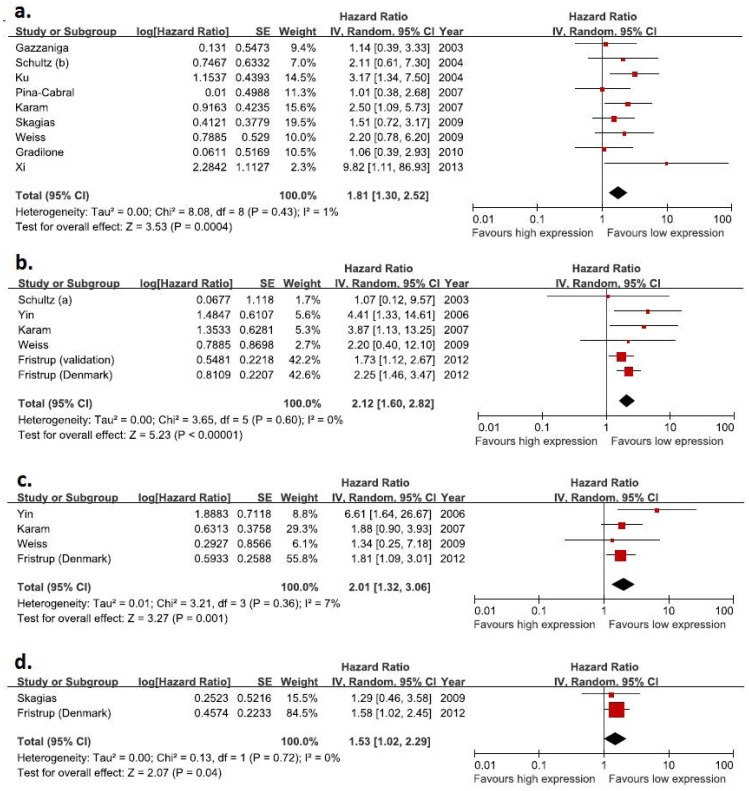
Forest plots of hazard ratios with random effects model for survivin in patients with non-muscle invasive bladder tumor. (A) Recurrence-free survival. (B) Progression-free survival. (C) Cancer-specific survival. (D) Overall survival.

Forrest plots of the primary meta-analyses can be seen in Fig. 2. Fig. 2 reports the average (pooled) HR and its 95% CI for each of the meta-analysis in muscle invasive bladder tumor. Each figure represents HR of survivin for recurrence-free survival (Fig. 2a), progression-free survival (Fig. 2b), and cancer-specific survival (Fig. 2c) and overall survival (Fig. 2d). The pooled HRs were statistically significant for recurrence-free survival (pooled HR, 1.81; 95% CI, 1.30–2.52), progression-free survival (pooled HR, 2.12; 95% CI, 1.60–2.82), cancer-specific survival (pooled HR, 2.01; 95% CI, 1.32–3.06), and overall survival (pooled HR, 1.53; 95% CI, 1.02–2.29).

In muscle invasive and advanced bladder tumors, the HRs were also statistically significant for recurrence-free survival (HR, 1.46; 95% CI, 1.18–1.82), cancer-specific survival (HR, 1.54; 95% CI, 1.21–1.96), and overall survival (HR, 2.46; 95% CI, 1.63–3.71). The results are shown in Fig. S1 and S2.

Only adjusted survival data were sufficient articles available to compare survival analyses according to survivin expression (Table 3), although this subgroup analysis only includes 2 studies with overall survival data available. Statistically significant differences were identified for all survival subgroup analyses. Survivin overexpression was significantly associated with adverse survival in the pooled patient group. In addition, sensitivity analyses confirm that our estimate of the overall HR of recurrence-free survival, progression-free survival, cancer-specific survival and overall survival by survivin status is robust when IHC was chosen for the method for determination of survivin expression (Table 4).

**Table 3:** Subgroup analysis in non-muscle invasive bladder tumor.

	No. of included articles	No. of cases	Pooled HR (95% CI)	I^2^	Chi^2^ (p value)
Recurrence-free survival	5*	368	2.09 (1.27–3.45)	27%	5.45 (0.24)
Progression-free survival	4**	868	2.17 (1.59–2.97)	8%	3.27 (0.35)
Cancer-specific survival	3†	458	2.17 (1.26–3.73)	33%	2.99 (0.22)
Overall survival	2‡	363	1.53 (1.02–2.29)	0%	0.13 (0.72)

HR: hazard ratio, CI: confidence interval.

* References: [11], [53], [56], [58], [61].

** References: [55,56,60 (Denmark cohort),60 (validation cohort)].

† References: [55,56,60 (Denmark cohort)].

‡ References: [58,60 (Denmark cohort)].

**Table 4:** Sensitivity analysis in non-muscle invasive bladder tumor.

	No. of included articles	No. of cases	Pooled HR (95% CI)	I^2^	Chi^2^ (p value)
Recurrence-free survival	5*	362	2.32 (1.53–3.52)	0%	3.52 (0.48)
Progression-free survival	5**	916	2.15 (1.62–2.86)	0%	3.27 (0.51)
Cancer-specific survival	4†	506	2.01 (1.32–3.06)	7%	3.21 (0.36)
Overall survival	2‡	363	1.53 (1.02–2.29)	0%	0.13 (0.72)

HR: hazard ratio, CI: confidence interval.

* References: [53], [56], [58], [59], [61].

** References: [55,56,59,60 (Denmark cohort),60 (validation cohort)].

† References: [55,56,59,60 (Denmark cohort)].

‡ References: [58,60 (Denmark cohort)].

Despite our attempts to limit between-study heterogeneity through our strict inclusion criteria, heterogeneity between overall survival results still remains within each subgroup and results should be interpreted cautiously.

**Figure 3:**
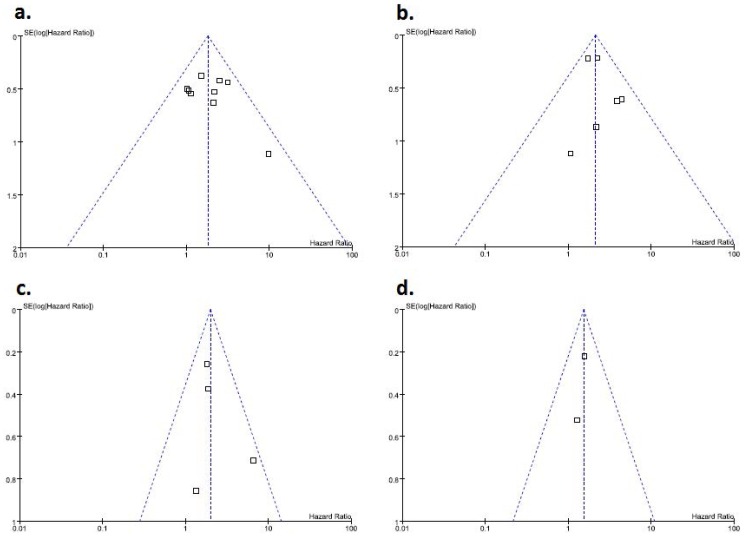
Funnel graphs of the assessment of potential publication bias in studies of survivin expression in patients with non-muscle invasive bladder tumor. (A) Recurrence-free survival. (B) Progression-free survival. (C) Cancer-specific survival. (D) Overall survival.

There was no clear evidence of funnel plot asymmetry for outcomes, and thus, there was no clear evidence of publication bias (Fig. 3). However, due to the small number of studies in most meta-analyses, it was not sensible to examine the potential for publication bias in meta-analysis, which did not contain 10 studies.

## Discussion

Currently, expression of survivin is being used as a novel prognostic factor in several human neoplasms. The rationale for investigating survivin as a prognostic marker in bladder cancer is based on its ability to inhibit apoptosis, promote proliferation and enhance angiogenesis, as well as its predominantly tumor-specific expression in adult tissues. In spite of suggested pivotal role of survivin as a prognostic marker, there are relatively few studies available exploring the role of survivin in bladder cancer, and some of them are controversial. In addition, the power of most individual studies was limited, due to low sample size. To date, no meta-analysis had been undertaken for any studies evaluating survivin as a prognostic marker in bladder cancer.

In this meta-analysis, which enrolled all the eligible studies comparing the survival of bladder cancer patients according to the tumor expression of survivin, survivin is a prognostic factor in bladder cancer. Our results showed that survivin overexpression is strongly predictive of recurrence, progression and mortality in bladder cancer.

Generally, meta-analysis based on individual data is considered as a gold standard [64]. However, meta-analysis of prognostic literature is associated with a number of inherent limitations. One of these key limitations is the general prevalence of retrospective study design in this setting. None of the studies included in the current meta-analysis specified a prospective design. It is difficult to draw any precise conclusions when studies are not conducted prospectively and when not all relevant data are available. Alongside this, an additional hindrance to meta-analysis of prognostic literature is the general lack of multivariable survival data in many of studies, although the REMARK guidelines state the investigation must include established clinicopathologic prognostic factors as part of a multivariate model, and report the resulting HRs regardless of statistical significance [14]. If the authors did not report the individual HR together with its variance, we calculated it from the survival comparison statistics and its variance, whenever possible. The estimated HR might be less reliable than the one obtained directly from published statistics. This is also attributable to the fact that the number of patients included in each study is typically small. However, when analyzing the overall relationship between individual study size and methological quality scores in the present study, there was no significant trend towards superior methodological quality in larger studies.

Although the specimens and methods used for the assessment of survivin expression in patients with bladder cancer differed among these studies, many of the eligible studies used IHC to detect survivin expression. IHC results should be interpreted with caution, because of varying specificity of the antibodies used, different concentration of the antibody used, lack of standardized technology, different approaches for storing and processing tissue, and the absence of a uniform definition of positive staining, leading to different results when using different cutoff points [65]. When defining survivin overexpression, the threshold in IHC varied from 8% to 20% among these studies. In patients with bladder cancer, there is no common threshold value in defining positive expression of survivin, but it is important that a common or standard threshold in the assessment of some biomarker should be set to make a comparatively accurate evaluation of its real function in clinical practice.

Survivin exists in two subcellular pools and this is consistent with its function in the regulation of both cell viability and cell division [66], [67]. Therefore, another problem with IHC is the determination of nuclear or cytoplasmic expression of survivin. Some studies pointed out the fact that survivin could be expressed in either cytoplasm or nuclei. For example, one study showed that survivin nuclear, but not cytoplasmic staining, correlated with tumor grade, stage, and patient outcome in patients with bladder cancer [55]. However, IHC results may sometimes lead to misjudgment or misinterpretation of the expression pattern of survivin in normal or cancerous tissues, due to inappropriate processing of either tissues or images [68]. In a review of the literature, Li et al [68] identified 19 publications that measured nuclear survivin in human tumors, and reported that conflicting findings existed on the relationship between nuclear survivin and prognosis. Among 19 publications, 9 showed that nuclear survivin expression is an unfavorable prognostic marker, whereas 5 proposed an opposing notion, i.e. that the nuclear survivin expression represented a favorable prognostic marker. The remaining 5 publications did not focus on studying the significance of survivin nuclear expression in disease outcome. Most eligible studies did not investigate the differential predictive value of nuclear versus cytoplasmic staining of survivin. At present, it remains uncertain as to whether there is a difference when distinguishing between cytoplasmic or nuclear staining for survivin.

Moreover, urine specimens were used to assess survivin mRNA in some studies [54], [57]. Since urine samples may contain variable numbers of tumor cells, the measured survivin levels might not truly represent tumor levels.

Although there was no heterogeneity for survival analysis, caution is perhaps advised, as there were only 14 studies with a relatively small sample size of patients in the analysis. Heterogeneity may be caused by other factors, such as inclusion criteria, different tumor stage, type of treatment, sample storage, primary antibody and dilution, method of measuring survivin, survivin cutoff levels, and adjustment for cofactors. It is also very difficult to examine or explain heterogeneity, due to the variability in clinical characteristics across patients within studies. In addition, there are few reports in the literature with respect to the prognostic impact of survivin in more advanced bladder cancer patients. Especially, only one study examined whether survivin overexpression might be a predictive marker for overall survival to cisplatin-based chemotherapy in patients with advanced (T4b and N2–N3) or metastatic (M1) bladder cancer [56].

Another potential source of bias is related to Language. This review was totally limited to literatures published in English because other languages were not accessible for the investigators. The restriction to English language articles possibly favors the positive results [69]. In addition, we did not extend the search to unpublished data that would likely include increased proportions of null results. Furthermore, the pooled risks of survivin for recurrence-free survivial or overall survival in non-muscle invasive bladder tumor, although statistically significant, were not strong, with pooled HRs of 1.81 and 1.53, respectively. Empirically, HR >2 is considered strongly predictive [70]. Finally, given the complexity of the molecular abnormalities associated with bladder cancer, combinations of independent, complementary markers might provide a more accurate prediction of outcome than a single marker [28], [50], [63].

Despite the inherent limitations of meta-analyzing prognostic literature, the findings from the present study suggest that survivin represents the consistently reproducible molecular marker with prognostic value in bladder cancer. Our strengths lie within the broad, unbiased search of the literature and the application of standardized systematic review and meta-analysis techniques to objectively identify manuscripts containing data sufficiently robust to be summarized. Strict inclusion/exclusion criteria were used to select the studies included in the present meta-analysis, thus limiting the potential bias. In cases where part or all of the same patients series was included in more than one publication, only the more recent or more complete study was included in the analysis, in order to avoid duplicating the same patient data. When considering the overall effects of potential publication bias in this analysis, the funnel plots for survival analysis were not indicative of any strong publication bias.

## Conclusions

In conclusion, our meta-analysis has yielded significant association between survivin expression and bladder cancer recurrence, progression, and mortality, although these findings need to be interpreted with caution. It is difficult to draw any reliable conclusion for the current meta-analysis of survivin for overall survival in bladder cancer, due to the limited number of evaluable studies. Survivin determination might help identify patients with bladder cancer at high risk of disease recurrence, progression and poor prognosis, who might benefit from closer follow-up or more aggressive therapy. However, simplified, quantitative and reproducible assays need to be developed and validated for the detection of survivin. In addition, it is rather necessary that better designed studies need to be enrolled into such kind of analysis in the future, to provide a better conclusion about the relationship between survivin expression and the outcome of patients with bladder cancer. The value of survivin for molecular staging of bladder cancer also needs to be confirmed in controlled trials involving larger number of patients with longer follow-up, before any definitive conclusions can be made.

## Supporting Information

Figure S1 **Forest plots of hazard ratios with random effects model for survivin in patients with muscle invasive bladder tumor.** (A) Recurrence-free survival. (B) Cancer-specific survival. (will be attached by *.TIF File)
(TIF)

Figure S2 **Forest plots of hazard ratios with random effects model for survivin in patients with advanced or metastatic bladder tumor (overall survival).** (will be attached by *.TIF File)
(TIF)

File S1 **Supporting tables.** Table S1. Patient characteristics. Table S2. Tumor characteristics. Table S3. Survivin expression according to pathological features.
(DOC)

Checklist S1 **PRISMA checklist part 1.**
(TIF)

Checklist S2 **PRISMA checklist part 2.**
(TIF)
