# Supplementary figures and images for: Prognostic Role of Survivin in Bladder Cancer: A Systematic Review and Meta-Analysis
Source: PLoS One. 2013 Oct 18;8(10):e76719. doi: 10.1371/journal.pone.0076719 (PMC3799942; doi:10.1371/journal.pone.0076719)

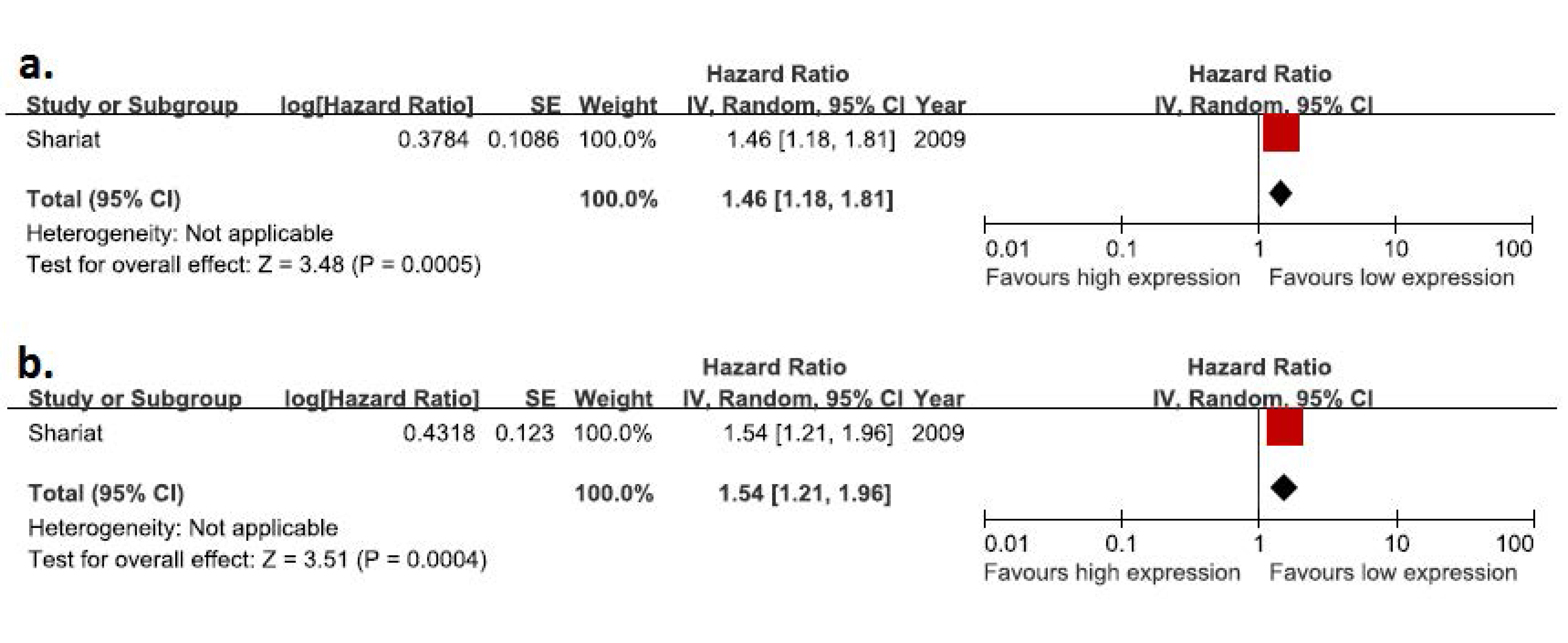

Supplement: Figure S1 — Forest plots of hazard ratios with random effects model for survivin in patients with muscle invasive bladder tumor. (A) Recurrence-free survival. (B) Cancer-specific survival. (will be attached by *.TIF File) (TIF) [file pone.0076719.s001.tif]

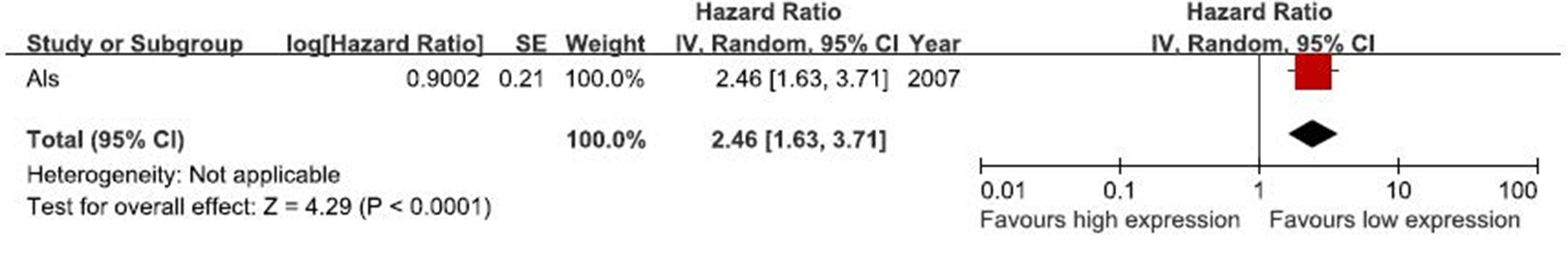

Supplement: Figure S2 — Forest plots of hazard ratios with random effects model for survivin in patients with advanced or metastatic bladder tumor (overall survival). (will be attached by *.TIF File) (TIF) [file pone.0076719.s002.tif]

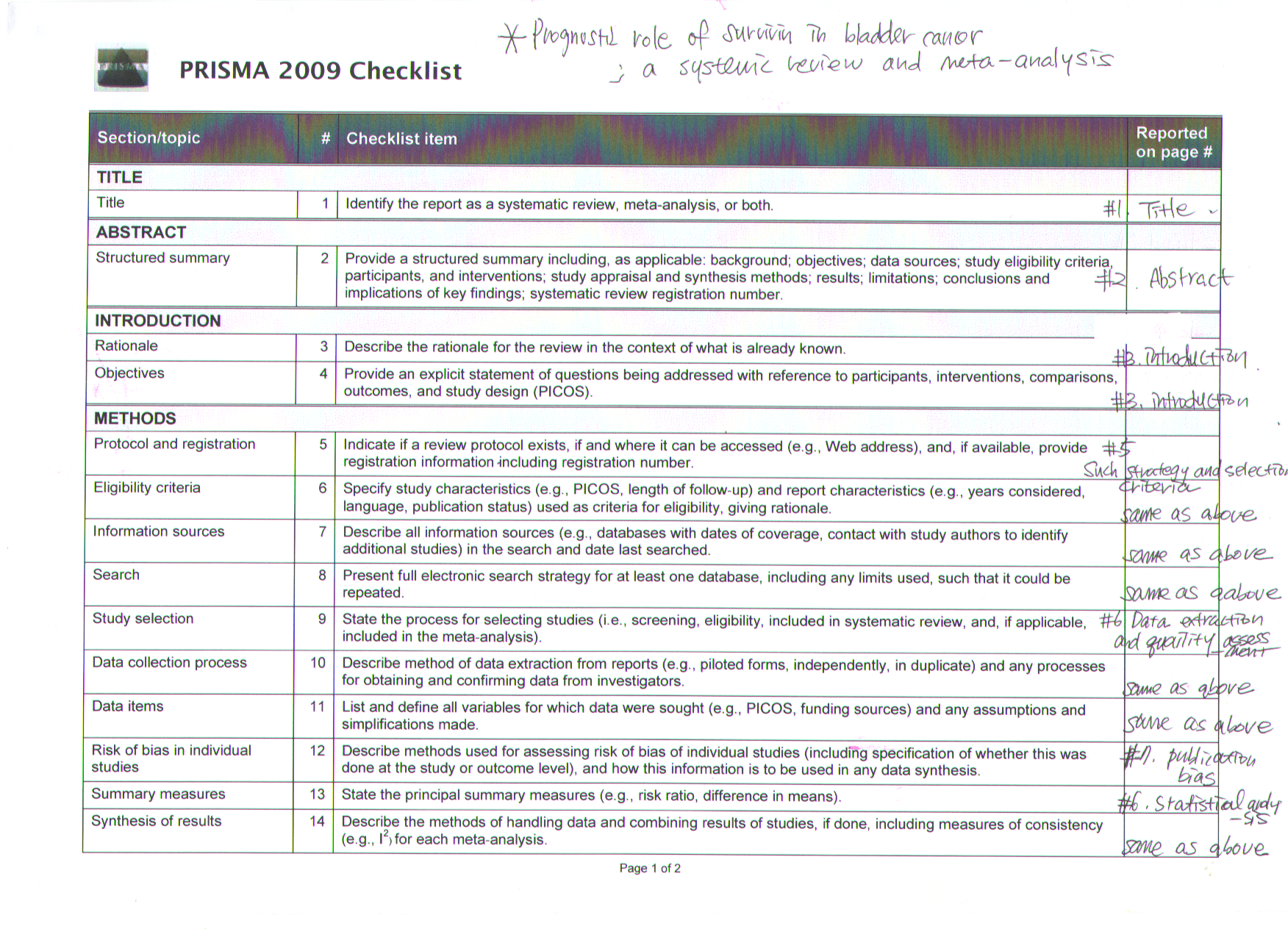

Supplement: Checklist S1 — PRISMA checklist part 1. (TIF) [file pone.0076719.s004.tif]

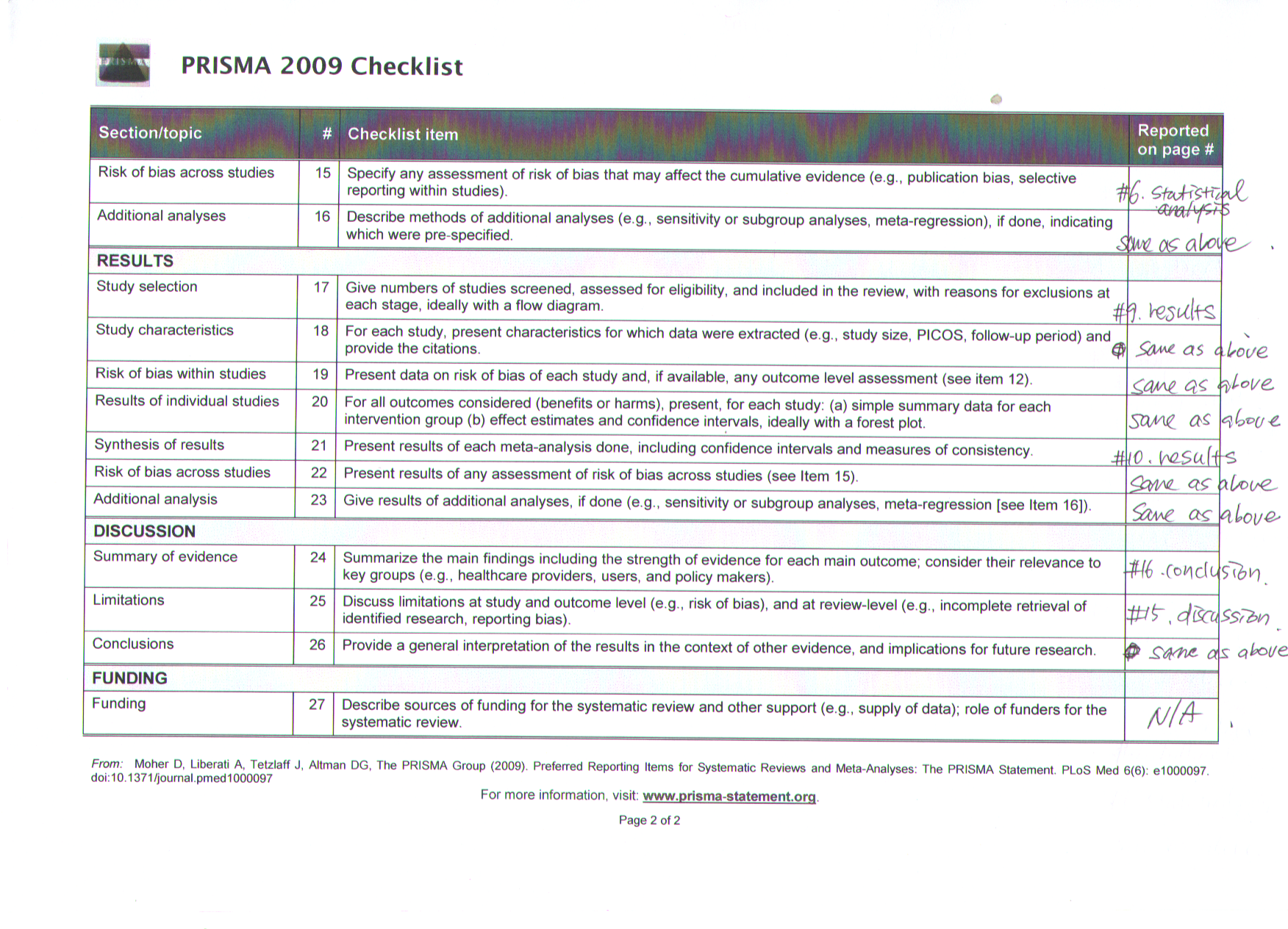

Supplement: Checklist S2 — PRISMA checklist part 2. (TIF) [file pone.0076719.s005.tif]
