# Supplementary material for: Prognostic Role of Survivin in Bladder Cancer: A Systematic Review and Meta-Analysis
Source: PLoS One. 2013 Oct 18;8(10):e76719. doi: 10.1371/journal.pone.0076719 (PMC3799942; doi:10.1371/journal.pone.0076719)
Supplement: File S1 — Supporting tables. Table S1. Patient characteristics. Table S2. Tumor characteristics. Table S3. Survivin expression according to pathological features. (DOC) [file pone.0076719.s003.doc]

Figure S1. Patient characteristics

| Study | No. of Patients | Median age, range (yr) | Gender (m/f) | Treatment | Adjuvant treatment | Median FU, range (mon) |
| --- | --- | --- | --- | --- | --- | --- |
| Gazzaniga^10^ | 30 | 65.0, 27-85 | NA | TURBT | 11 (intravesical MMC)  7 (intravesical BCG) | 39.0 (mean), 27-51 |
| Schultz^52^ | 17 | NA | NA | TURBT | 17 (intravesical) | 70.8 (mean), 2-180 |
| Ku^53^ | 88 | 60 (mean), 23-92 | 80/8 | TURBT | NA | 63, 1-113 |
| Schultz^54^ | 26 | NA | NA | TURBT | 13 (intravesical) | 32.6 (mean), 1-45 |
| Yin^55^ | 101 | NA | 81/20 | TURBT | 101 (intravesical BCG) | 54, 20-68.6 (10-90% percentiles) |
| Karam^56^ | 74 | 63.2, 41.1-89.3 | 60/14 | TURBT | 54 (intravesical MMC or BCG) | 42.3, 0.3-124.6 |
| Pina-Cabral^57^ | 30 | 74.5, 39-86 | 23/7 | TURBT | 17 (intravesical MMC or BCG) | 22.3, 2.8-41.4 |
| Skagias^58^ | 80 | 65 (mean), 26-85 | 69/11 | TURBT or radical cystectomy | NA | 33.9 (mean), 12-96 |
| Weiss^59^ | 48 | 71, NA | 40/8 | TURBT | 8 (RT), 40 (CRT) | 27.0, 3-140 |
| Gradilone^11^ | 54 | 57.5, 51-64 | NA | TURBT | 54 (intravesical BCG) | 17.9 (mean), 3-24 |
| Fristrup (Denmark)^60^ | 283 | 68, 32-86 | 222/61 | TURBT | 70 (intravesical MMC or BCG) | 103, 2-263 |
| Fristrup (validation1)^60^ | 141 | 70, 31-96 | 112/29 | TURBT | NA | 72, 1-193 |
| Fristrup (validation2)^60^ | 269 | 68, 25-89 | 233/36 | TURBT | 193 (intravesical MMC or BCG) | 99, 3-205 |
| Xi^61^ | 72 | NA | 59/13 | TURBT | 61 (intravesical or systemic chemotherapy) | 51 (mean), 21-60 |
| Shariat^62^ | 726 | 68, 34-94 | 600/126 | radical cystectomy | 187 (systemic chemotherapy) | 53.3, 0.1-235.6 |
| Als^63^ | 25 (microarray),  101 (IHC) | 51.5, 49-74 (microarray),  62.6 (31-78 (IHC) | 24/6 (microarray), 96/28 (IHC) | systemic chemotherapy | 11 (RT or surgery) | 81.8, 56.7-98.0 (microarray),  56.5, 19.5-129.8 (IHC) |

FU: follow-up, NA: not available, TURBT: transurethral resection of bladder tumor, MMC: mitomycin C, BCG: bacillus Calmette-Guérin, RT: radiotherapy, CRT: chemoradiotherapy, IHC: immunohistochemistry.

Figure S2. Tumor characteristics

| Study | T stage  (Ta/Tis/T1) | Concomitant CIS | Tumor grade  (G1/G2/G3) | Multiplicity  (single/multiple/NA) | Tumor architecture  (papillary/solid/mixed) | Tumor size  (<3cm/≥3cm/NA) | Positive survivin expression |
| --- | --- | --- | --- | --- | --- | --- | --- |
| Gazzaniga^10^ | 9/0/21 | NA | 21/9/0 | 17/13/0 | NA/NA/NA | NA/NA/NA | 9 |
| Schultz^52^ | NA/NA/NA | NA | NA/NA/NA | NA/NA/NA | NA/NA/NA | NA/NA/NA | 8 |
| Ku^53^ | 44/0/44 | NA | 20/47/21 | 48/40/0 | 79/9/0 | 75/13/0 | 51 |
| Schultz^54^ | NA/NA/NA | NA | NA/NA/NA | NA/NA/NA | NA/NA/NA | NA/NA/NA | 13 |
| Yin^55^ | 54/0/47 | 0 | 59 (LG)/42 (HG) | NA/NA/NA | NA/NA/NA | NA/NA/NA | 28 |
| Karam^56^ | 26/13/35 | NA | 7/32/35 | NA/NA/NA | NA/NA/NA | NA/NA/NA | 39 |
| Pina-Cabral^57^ | 15/2/13 | NA | 16/10/4 | 14/16/0 | NA/NA/NA | NA/NA/NA | 20 |
| Skagias^58^ | 51/0/15/14 (≥T2) | NA | 52 (LG)/28 (HG) | NA/NA/NA | NA/NA/NA | NA/NA/NA | 49 |
| Weiss^59^ | 0/0/48 | 14 | 12 (G12)/36 | 24/24/0 | NA/NA/NA | NA/NA/NA | 32 |
| Gradilone^11^ | 0/0/54 | 0 | 0/0/54 | 54/0/0 | NA/NA/NA | 54/0/0 | 27 |
| Fristrup (Denmark)^60^ | 182/0/101 | 95 | 183 (LG)/100 (HG) | 169/87/27 | 248/20/14 | 177/72/34 | 98 |
| Fristrup (validation1)^60^ | 67/0/74 | NA | 47 (LG)/94 (HG) | NA/NA/NA | NA/NA/NA | NA/NA/NA | NA |
| Fristrup (validation2)^60^ | 21/0/248 | 70 | 103 (LG)/166 (HG) | NA/NA/NA | 230/22/17 | 187/78/4 | NA |
| Xi^61^ | 25 (TaTis)/47 | NA | 41 (G12)/31 | NA/NA/NA | NA/NA/NA | NA/NA/NA | 61 |
| Shariat^62^ | 90 (T1)/208 (T2)/309 (T3)/119 (T4) | NA | 108 (LG)/618 (HG) | NA/NA/NA | NA/NA/NA | NA/NA/NA | 359 |
| Als^63^ | 124 (≥T4b) | NA | NA/NA/NA | NA/NA/NA | NA/NA/NA | NA/NA/NA | 52 |

CIS: carcinoma in situ, NA: not available, LG: low grade, HG: high grade.

Figure S3. Survivin expression according to pathological features

|  | T stage | | | Concomitant CIS | Tumor grade | | | Multiplicity | | | Tumor architecture | | | Tumor size | | |
| --- | --- | --- | --- | --- | --- | --- | --- | --- | --- | --- | --- | --- | --- | --- | --- | --- |
|  | Ta | Tis | T1 |  | G1 | G2 | G3 | single | multiple | NA | papillary | solid | mixed | 3cm | ≥3cm | NA |
| Gazzaniga^10^ | 1/9 | 0/0 | 8/21 | NA | 1/21 | 8/9 | 0/0 | 6/17 | 3/13 | 0/0 | NA | NA | NA | NA | NA | NA |
| Schultz^52^ | NA | NA | NA | NA | NA | NA | NA | NA | NA | NA | NA | NA | NA | NA | NA | NA |
| Ku^53^ | 22/44 | 0/0 | 29/44 | NA | 35/67 (G12) | | 16/21 | 27/48 | 24/40 | 0/0 | 47/79 | 4/9 | 0/0 | 43/75 | 8/13 | 0/0 |
| Schultz^54^ | NA | NA | NA | NA | NA | NA | NA | NA | NA | NA | NA | NA | NA | NA | NA | NA |
| Yin^55^ | NA | NA | NA | NA | NA | NA | NA | NA | NA | NA | NA | NA | NA | NA | NA | NA |
| Karam^56^ | 11/26 | 9/13 | 19/35 | NA | 2/7 | 10/32 | 27/35 | NA | NA | NA | NA | NA | NA | NA | NA | NA |
| Pina-Cabral^57^ | 9/15 | 2/2 | 9/13 | NA | 8/16 | 8/10 | 4/4 | 11/14 | 9/16 | 0/0 | NA | NA | NA | NA | NA | NA |
| Skagias^58^ | 37/66 (≤T1), 12/14 (≥T2) | | | NA | 25/52 (LG), 24/28 (HG) | | | NA | NA | NA | NA | NA | NA | NA | NA | NA |
| Weiss^59^ | 0/0 | 0/0 | 32/48 | 8/14 | 8/12 (G12) | | 24/36 | 15/24 | 17/24 | 0/0 | NA | NA | NA | NA | NA | NA |
| Gradilone^11^ | NA | NA | NA | NA | NA | NA | NA | NA | NA | NA | NA | NA | NA | NA | NA | NA |
| Fristrup (Denmark)^60^ | 51/182 | 0 | 47/101 | 40/95 | 49/183 (LG), 49/100 (HG) | | | 55/169 | 36/87 | 7/27 | 83/248 | 7/20 | 8/14 | 61/177 | 28/72 | 9/34 |
| Fristrup (validation1)^60^ | NA | NA | NA | NA | NA | NA | NA | NA | NA | NA | NA | NA | NA | NA | NA | NA |
| Fristrup (validation2)^60^ | NA | NA | NA | NA | NA | NA | NA | NA | NA | NA | NA | NA | NA | NA | NA | NA |
| Xi^61^ | 19/25 (TaTis) | | 42/47 | NA | 31/41 (G12) | | 30/31 | NA | NA | NA | NA | NA | NA | NA | NA | NA |
| Shariat^62^ | 44/90 (T1), 81/208 (T2),  162/309 (T3), 72/119 (T4) | | | NA | 59/108 (LG), 300/618 (HG) | | | NA | NA | NA | NA | NA | NA | NA | NA | NA |
| Als^63^ | NA | NA | NA | NA | NA | NA | NA | NA | NA | NA | NA | NA | NA | NA | NA | NA |

CIS: carcinoma in situ, NA: not available, LG: low grade, HG: high grade.
